# Cytostatic and Cytotoxic Natural Products against Cancer Cell Models

**DOI:** 10.3390/molecules24102012

**Published:** 2019-05-26

**Authors:** Taotao Ling, Walter H. Lang, Julie Maier, Marizza Quintana Centurion, Fatima Rivas

**Affiliations:** 1 Department of Chemical Biology and Therapeutics, St. Jude Children’s Research Hospital. 262 Danny Thomas Place. Memphis, TN 38105-3678, USA; Taotao.Ling@STJUDE.ORG (T.L.); Walter.Lang@STJUDE.ORG (W.H.L.); Julie.Maier@STJUDE.ORG (J.M.); 2 Dirección de Investigación Biológica/Museo Nacional de Historia Natural del Paraguay, Casilla de Correo 19.004. Sucursal 1, Campus UNA. 169 CDP San Lorenzo, Central XI, Paraguay; bmquintana2008@hotmail.com

**Keywords:** natural product alkaloids, cephalotaxine, protein synthesis inhibition, antiproliferation agents, cancer

## Abstract

The increasing prevalence of drug resistant and/or high-risk cancers indicate further drug discovery research is required to improve patient outcome. This study outlines a simplified approach to identify lead compounds from natural products against several cancer cell lines, and provides the basis to better understand structure activity relationship of the natural product cephalotaxine. Using high-throughput screening, a natural product library containing fractions and pure compounds was interrogated for proliferation inhibition in acute lymphoblastic leukemia cellular models (SUP-B15 and KOPN-8). Initial hits were verified in control and counter screens, and those with EC_50_ values ranging from nanomolar to low micromolar were further characterized via mass spectrometry, NMR, and cytotoxicity measurements. Most of the active compounds were alkaloid natural products including cephalotaxine and homoharringtonine, which were validated as protein synthesis inhibitors with significant potency against several cancer cell lines. A generated BODIPY-cephalotaxine probe provides insight into the mode of action of cephalotaxine and further rationale for its weaker potency when compared to homoharringtonine. The steroidal natural products (ecdysone and muristerone A) also showed modest biological activity and protein synthesis inhibition. Altogether, these findings demonstrate that natural products continue to provide insight into structure and function of molecules with therapeutic potential against drug resistant cancer cell models.

## 1. Introduction

**Figure 1:**
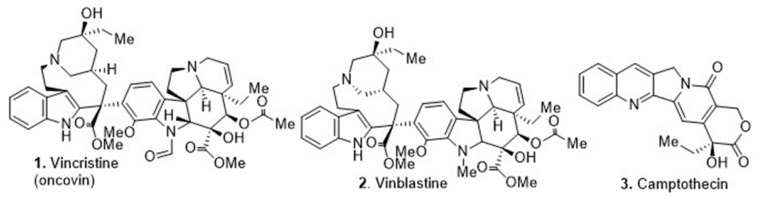
Natural product-derived alkaloids in clinical use.

Cancer is a complex chronic disease characterized by abnormal signaling processes that leads to aberrant cellular growth, causing premature death worldwide [1,2]. Natural products have a strong track record in the development of anti-cancer agents, thus many drug discovery programs continue to exploit this rich source of molecular scaffolds [3]. The re-emergence of natural products for drug discovery in the genomics era enables the use of advanced cellular models that recapitulate the disease of interest. Natural products, particularly alkaloids, are commonly used in ethnopharmacology and several are in clinical use (Figure 1). 

Acute lymphoblastic leukemia (ALL) is among the most common pediatric cancers, occurring in approximately 1:1500 children [4]. Specific genetic aberrations define B cell precursor ALL subtypes with distinct biological and clinical characteristics. A class of genetic aberrations comprises tyrosine kinase-activating lesions, including translocations and rearrangements of tyrosine kinase and cytokine receptor genes such as the Philadelphia chromosome (Ph/BCR/ABL+) lesion among other genetic abnormalities leading to drug resistance. While response to high-risk drug treatment varies, drug resistance or disease recurrence are responsible for frequent causes of treatment failure [4,5,6]. 

The application of cellular screening systems and technological advancements in cell and molecular biology enable the chain of translatability by using disease-relevant cell models that display a more truthful phenotype, therefore aiding in the identification of new molecular scaffolds with clinical potential. Following this paradigm, the main focus of this study was to identify compound hits against high-risk cellular models of ALL (SUP-B15 and KOPN-8) while displaying therapeutic index (TI) when compared to normal cells (BJ and PBMCs), using an enriched library of natural product fractions [7,8,9,10] from a subgroup of alkaloid-producing plants (Annonaceae, Aristolochiaceae, Berberidaceae, Eupomatiaceae Fabaceae, Fumariaceae, Lauraceeae, Magnoliaceae, Monimiaceae, Nelumbonaceae, Papaveraceae, Ranunculaceae, Combretaceae, Rutaceae, Araliaceae, Apiaceae, Rubiaceae, and Araceae families), which were collected in collaboration with Museo Nacional de Historia Natural del Paraguay where several of these specimens were collected and have been deposited. 

## 2. Results

### 2.1. Cytostatic/Cytotoxic Evaluation via Cell Proliferation Assay

**Figure 2:**
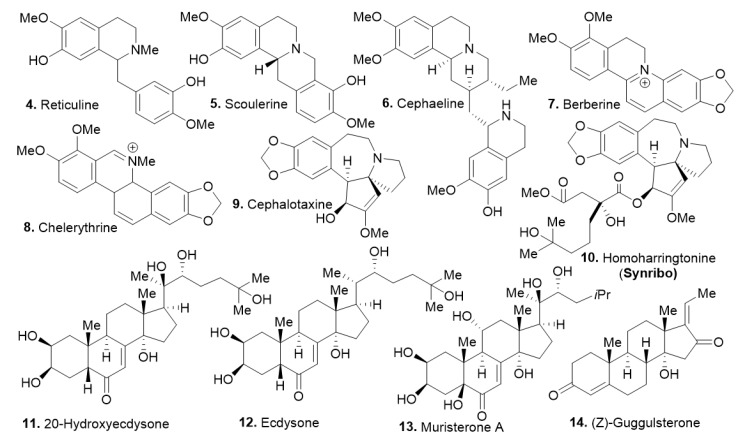
Identified natural products with cytostatic and cytotoxicity activity against ALL cell lines.

A natural product library containing fractions (3K) and pure compounds (2K) were evaluated in a single point cell proliferation assay (CTG), as established in our group [11,12,13], and the Z’ values were consistently higher than 0.5, revealing a large separation between positive and negative controls (Supplementary Material, Figures S1 and S2). From the single point primary screen, 22 compounds made the cutoff of 50% inhibition at 100 µM. Then, the most potent fractions were validated by dose-response CTG assay and the corresponding compounds elucidated by NMR and mass spectrometry (Figure 2). The biological activities of the pure natural products were confirmed and EC_50_ values are shown in Table 1 (Supplementary Material, Figures S3 and S4), providing several compounds with promising anti-proliferative effects against some of the high-risk cancer cell models disclosed in this work. 

The identified compounds exhibit half maximal effective concentration (EC_50_) in the low micromolar range (<10 µM) against cancer cell lines, while displaying low or no cytotoxicity against non-cancerous cells (therapeutic index, TI > 5). Assessment in a broad range of non-cancerous tissue provides insightful information, thus BJ and PBMC cells were selected to determine TI (a higher TI is desired). Several leukemia cellular models were utilized for the viability assay to evaluate the scope of activity, but the study was particularly focused on a subset of ALL, namely KOPN-8 and SUP-B15 models, which carry specific genomic lesions. KOPN-8 carries the MLL-ENL fusion gene and SUP-B15 was established from a pediatric ALL relapsed patient (with the m-BCR ALL variant of the BCR-ABL1 fusion gene) [14].

The gene for the histone methyltransferase (MLL) participates in chromosomal translocations that eventually create MLL-fusion proteins associated with very aggressive forms of childhood acute leukemia, which serve as an independent dismal prognostic factor for this patient cohort [4,5,6]. Therefore, the identification of compounds against models with MLL gene fusions can provide insight into the discovery of new therapies. The chemical treatment response was similar for the ALL cell models, as shown in Table 1. Further evaluation demonstrated the most active compounds induced cell death in a concentration- dependent manner.

The identified compounds share properties with ample opportunity for improvement and further formulation to improve the compounds’ physicochemical properties and suitability for specific delivery methods. While the compounds show similar antiproliferative activities, no distinctive structural similarities were recorded for the most potent compounds other than their shared alkaloid core between **4**–**10**, and the steroidal core for compounds **11**–**14**. Among these compounds, homoharringtonine (compound **10** [15,16,17]), a known potent protein synthesis inhibitor already approved for clinical use by the United States Food and Drug Administration against chronic myeloid leukemia (CML) [18,19,20] was identified. It shows high potency in the pre-B ALL cell models (SUP-B15 and KOPN-8) in agreement with previous reports [21,22]. However, it was not clear whether cephalotaxine (compound **9**) worked via the same mechanism as compound **10**. While the steroidal compounds (**11**–**14**) displayed much weaker potency, it was important to further evaluate their potential against these cell lines. Under these experimental conditions, compound **10** showed the best potency against SUP-B15, NALM-06, and UoC-B1 (a glucocorticoid resistant cell line) with an EC_50_ of 0.0452 µM, 0.0322 µM, and 0.0129 µM, respectively, with no observable activity against BJ at the highest tested concentration (43.3 µM). Overall, the natural product fractions library provided molecular scaffolds with specific cell death mechanisms that renders acceptable therapeutic index as observed herein. 

### 2.2. Cell Cycle Arrest and Apoptosis 

To determine if cell cycle progression was affected by the most active compounds **8**–**13**, KOPN-8 and SUP-B15 cancer cells were investigated. Silvestrol, a protein synthesis inhibitor currently under clinical trials which exhibits significant cytotoxic activity against several human cancer cell lines such as oral carcinoma, melanoma, acute myelogenous leukemia, and cervical cancer with IC_50_ values in the low micromolar range [23,24,25,26], was used as a positive control. 

**Figure 3:**
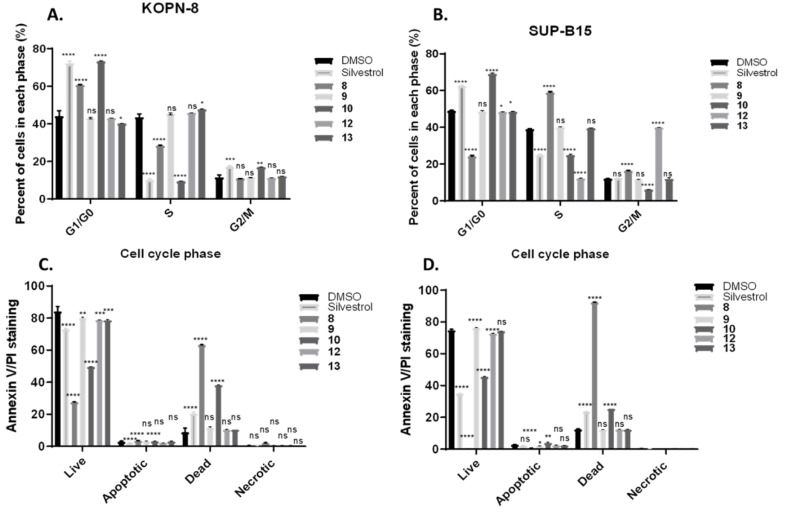
Analysis of apoptosis and cell cycle of compounds **8**–**10**, **12**, and **13** by Annexin V/Propidium Iodide (PI) flow cytometry assay after 24 h treatment using KOPN-8 and SUP-B15 cellular models. Negative control (DMSO), positive control (silvestrol, 5 μM), compounds **8** (5 μM), **9** (10 μM), **10** (5 μM), **12** (10 μM), **13** (10 μM). **A.** KOPN-8. **B.** SUP-B15. **C.** Annexin V/PI of KOPN-8. **D.** Annexin V/PI of SUP-B15. Bars depict mean and SD of at least three independent experiments. **** *p* < 0.0001, *** *p* < 0.0004, ** *p* < 0.0086, * *p* < 0.025 and ns (no statistical significant) according to Tukey’s test when compared to DMSO control.

Proliferating cells proceed through various phases of the cell cycle (G0, G1, S, G2, and M phase), and protein synthesis inhibitors can regulate cell cycle and cell proliferation. No serum starvation or phase-synchronization methods were used for these cells, in order to avoid secondary effects due to such manipulation [27,28]. The DMSO control indicates that both cell lines were primarily at the G0/G1 and S phase at the time of the experiment and only a small number (10%) were entering the G2/M phase (Supplementary Material, Figures S7 and S8). Silvestrol showed cell arrest in the G1/G0 phase of both cell lines. While compound **8** displayed a significant cell arrest in G1/G0 in KOPN-8, the cell arrest was more significantly increased in the percentage of cells (~20%) in S phase, with a significant reduction in the percentage of cells (~10%) in G2/M phase of SUP-B15. Interestingly, compound **10**’s profile was almost identical to silvestrol at the same concentration (5 µM) in both cell lines. However, cephalotaxine, which had shown antiproliferation effects by CTG at 10 µM, showed no significant cell arrest. Compounds **12** and **13** had minimal effect on the cell cycle in KOPN-8, with only a small effect in the S phase by compound **13**. Conversely, compound **12** arrested G2/M with a significant reduction in the percentage of cells (~10%) in the S phase in the SUP-B15 cell line (Figure 3A, B). Similar results were obtained in colorectal and hepatocellular cancer cells treated with silvestrol, where most cells were stalled in the early stages of the cycle [26]. Since bypass of the G1 phase of the cycle due to DNA damage leads to apoptosis, to determine whether the compounds induce programmed cell death in KOPN-8 and SUP-B15, the cells were double stained with Annexin V and PI dyes to determine the percentage of cells in early vs late apoptosis and viable cells (Supplementary Material, Figures S5 and S6). As shown in Figure 3C, D, there is a significant decrease in live cells for compounds **8**–**10** in KOPN-8 and SUP-B15. Most of the cells treated by compound **8** were not viable, while treatment with compound **9** only shows a small decrease in live cells (Annexin-, PI-), accompanied with an increase in apoptotic cells or dead cells during the 24 h treatment. Data presented on compounds **12** and **13** did not capture significant increase of late apoptotic state or cell death upon treatment for either cell line, but there was a small decrease in live cells by compound **12**. These results in combination with viability data (CTG) suggests that these steroidal compounds (**11**–**14**) are likely cytostatic agents and should be more effective in combination therapy. Data are shown from at least three independent experiments and statistical analysis of data was performed using Graph Pad Prism 7. The differences between the groups and negative control were analyzed by Tukey’s test, with standard error bars representing the standard deviation of the mean (± SD).

**Figure 4:**
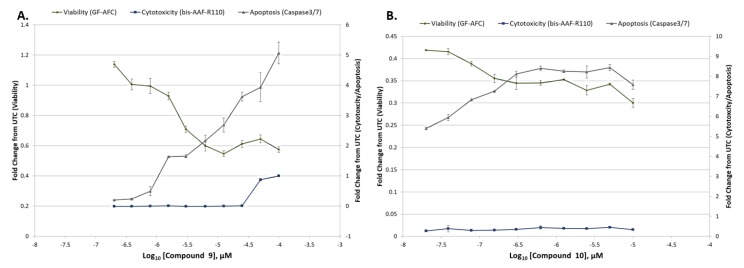
The ApoTox-Glo triplex assay against SUP-B15 to determine viable cells (using GF-AFC as fluorescent readout), apoptotic cells (caspase 3/7Glo as luminescent readout) or cytotoxicity by membrane integrity (bis-AAF-RF110 as fluorescent readout) upon compound treatment for 36 h. **A.** compound **9** (0.2–100 µM). **B.** compound **10** (0.02–10 µM). Graphs show decrease in cell viability, while increasing apoptotic activity with little cytotoxic effects by membrane integrity evaluation under the evaluated conditions (time and concentration).

In addition to Annexin V staining for apoptosis induction, an alternate method of apoptosis detection method such as caspase 3/7 activity assay was performed. To validate these results, ApoTox-Glo triplex assay (Promega) was performed to determine viable cells (GF-AFC), apoptotic cells (caspase 3/7), or cytotoxicity by membrane integrity (bis-AAF-RF110) as an alternative cell death modality induced by compounds **9** and **10**. Both compounds increased caspase 3/7 activity, and decreased viability of SUP-B15 cells, further validating the cell death inducing effects of these compounds (Figure 4). The data indicate that compounds **9** and **10** might undergo similar cell death mechanisms, albeit at different concentrations, and suggests apoptosis is dependent on caspase activity in this cell line [29]. 

**Figure 5:**
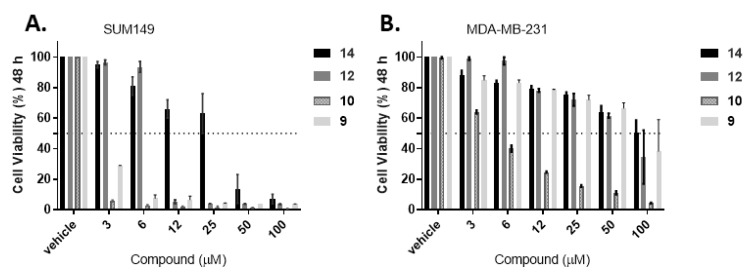
Propidium iodide (PI) assay of compounds **9**–**10**, **12** and **14** after 48 h treatment against breast cancer cell models to determine apoptotic effects. **A.** SUM-149, and **B.** MDA-MB-231. Bars represent mean ± SEM of at least three biological replicates.

To conduct live cell imaging studies (protein synthesis and co-localization studies), the adherent triple negative breast cellular models were selected as such studies are currently not feasible with suspension cells (leukemia cells). The first step was to evaluate if the compounds display cytotoxicity against the adherent cells. Propidium iodide (PI) assay was performed for the drug resistant solid tumor cellular models (triple negative breast cancer models: SUM149 and MDA-MB-231) for 48 h (Figure 5) [30,31]. All the tested compounds had better efficacy against SUM149 with promising activity at 12 µM, except for compound **14**, which displayed the weakest activity against the tested cancer cell lines. Compound **9** demonstrated 50% reduction of cell viability even at 3 µM concentration in SUM149, while only compounds **9** and **10** showed substantial activity in MDA-MB-231 cell line (drastically reducing cell viability at the highest concentration).

### 2.3. Protein Synthesis Evaluation

**Figure 6:**
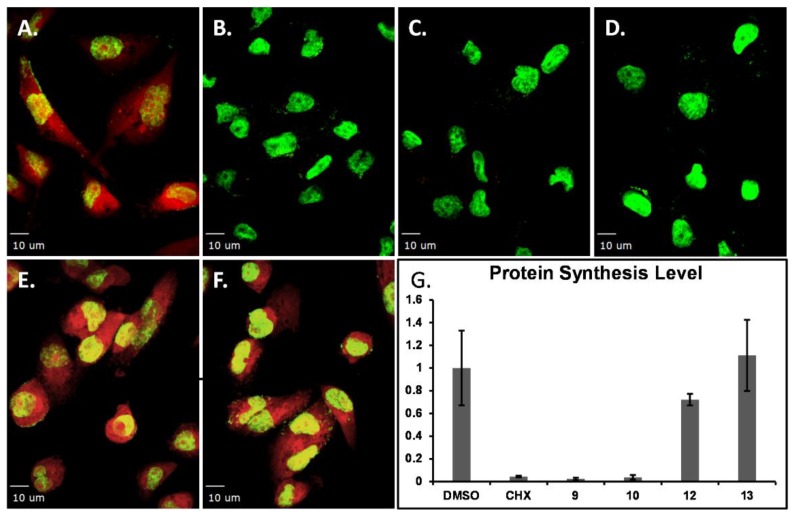
Representative images of protein synthesis inhibition EZClick™ assay using MDA-MB-231 cellular model. Cells were treated with compounds for 1.5 h prior to click reaction followed by staining. **A.** Vehicle (DMSO). **B.** Positive control **CHX** (1 μM). **C.** Compound **9** (5 μM). **D.** Compound **10** (10 μM). **E.** Compound **12** (10 μM). **F.** Compound **13** (10 μM). **G.** Relative quantification. Scale bar: 10 μm.

Protein synthesis is essential in cell growth, proliferation, signaling, differentiation, and death [32]. To interrogate whether the compounds inhibited protein synthesis as nascent proteins are generated, protein synthesis levels were monitored using a commercially available kit (EZClick™ Global Protein Synthesis Assay Kit). The assay includes a robust chemical method based on an alkyne containing o-propargyl-puromycin probe, which stops translation by forming covalent conjugates with nascent polypeptide chains. Truncated polypeptides are rapidly turned over by the proteasome and can be detected based on the subsequent click reaction with a fluorescent azide [33]. To test whether these compounds (**4**–**14**) induced de novo protein synthesis inhibition, compounds were tested for a 2 h exposure time, under the same conditions as the known protein synthesis inhibitor cycloheximide (CHX) in MDA-MB-231 cell model following an in-cell-click de novo protein synthesis assay [33,34,35]. Partial protein synthesis inhibition was observed for compound **4** (Supplementary Material, Figure S9), and compounds **7**–**8** showed no inhibition (data not shown). The results show that compound **9** inhibits protein synthesis at 10 µM while compound **10** inhibits de novo protein synthesis at lower concentrations (1–5 µM, higher concentrations induce immediate cell detachment) under these experimental conditions (Figure 6A, B). Compounds **12** and **13** showed little to no protein synthesis inhibition, as shown in Figure 6E–G along with their relative quantification. The remaining compounds (**11** and **14**) showed no protein synthesis inhibition. 

### 2.4. Probe Synthesis and Evaluation 

**Scheme 1:**
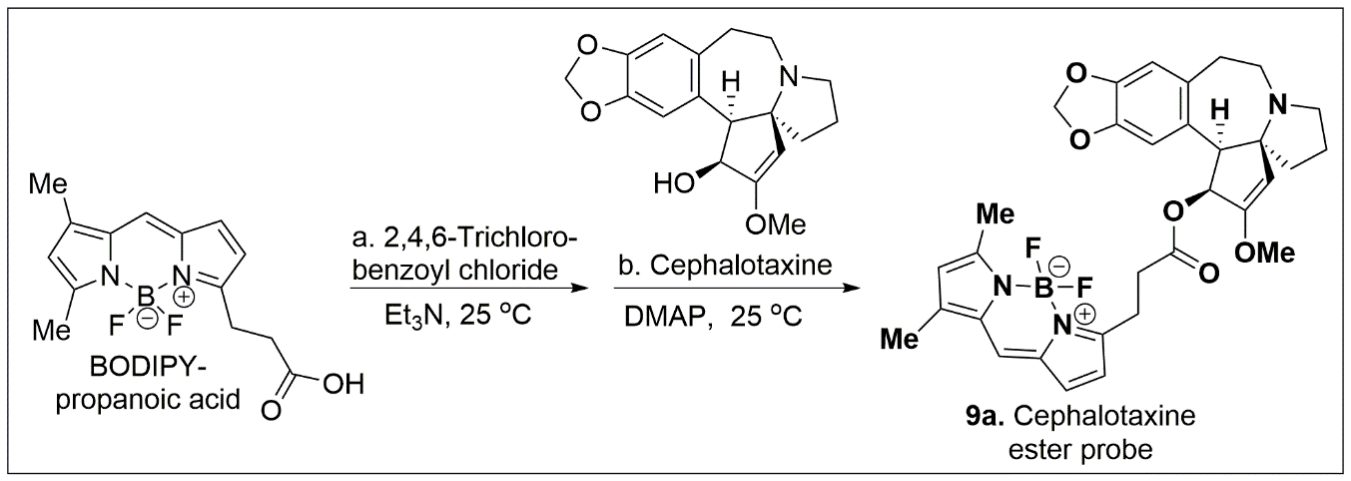
Synthesis of compound **9a**. (a) 2.0 equiv Et_3_N, 25 °C, 1 h; (b) 1. 0 equiv cephalotaxine 0.1 equiv DMAP, DCM, 25 °C, 16 h.

Fluorescent labels are generally used in bio-orthogonal labelling for co-localization studies in order to better understand the compounds’ mode of action [36]. Small-molecule fluorophores are the dominant method of choice due to their relative ease of use and excellent sensitivity, together with good spatial and temporal resolution. Washout studies of the corresponding BODIPY-FL ester have reliably shown that the reagent does not accumulate in the cell. Thus, 3-BODIPY-FL was treated with 2,4,6-trichlorobenzoyl chloride and Et_3_N for 1 h in DCM, followed by addition of cephalotaxine along with DMAP in DCM for 16 h RT to provide probe **9a** in 87% overall yield as shown in Scheme 1 (Supplementary Material, Figures S11–S14). 

**Figure 7:**
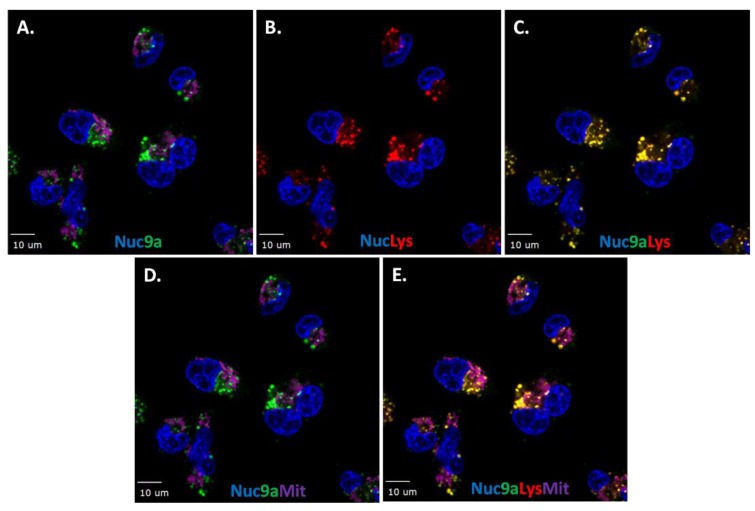
Representative images of co-localization studies of probe **9a** with organelle fluorescent trackers in live MDA-MB-231 cells. **A.** Hoechst nuclear stain (blue) and probe **9a** (1 µM, green). **B.** Lysosome tracker (red) and nuclear stain (blue). **C.** Merge of probe **9a**, lysosome and nuclear stains. **D.** MitoTracker Deep Red (purple), nuclear stain (blue) and compound **9a** (green). **E.** Nuclear stain, probe **9a**, Lysosome (red) and MitoTracker Deep Red. Scale bar: 10 μm.

It is important to determine the intracellular accumulation of compound **9** as this may aid in identifying its intracellular interactions. To facilitate these experiments, a series of orthogonal fluorescent organelle markers were used for co-localization studies. The organelle marker set included indicators for the lysosome (Lysotracker Red), the mitochondria (MitoTracker Deep Red), and the endoplasmic reticulum (ER tracker Blue/White). Where applicable, nuclei were stained with Hoechst (Blue). All these markers were compatible with BODIPY-FL (green) to allow for flexible combinations. The MDA-MB-231 adherent cellular model was used for the live cell co-localization studies as this cellular model displays well-defined organelle morphologies that can be consistently and positively identified [37]. First, to evaluate the accumulation of compound **9a** after 30 min treatment at 1 µM, washout experiments were conducted as shown in Figure 7A in the presence of Hoechst nuclear stain. Distinct green specks were observed near the nucleus of the cell. A similar observation was made using lysosome tracker (red, Figure 7B) and merging of both images clearly shows co-localization, observed as yellow (Figure 7C). Next MitoTracker-Deep Red (purple) was evaluated (Figure 7D) but no co-localization was detected with probe **9a** as seen by the absence of white staining in the merged image. This demonstrates that probe **9a** co-localizes with lysosomes, but not with mitochondria inside the cell (Figure 7E). 

**Figure 8:**
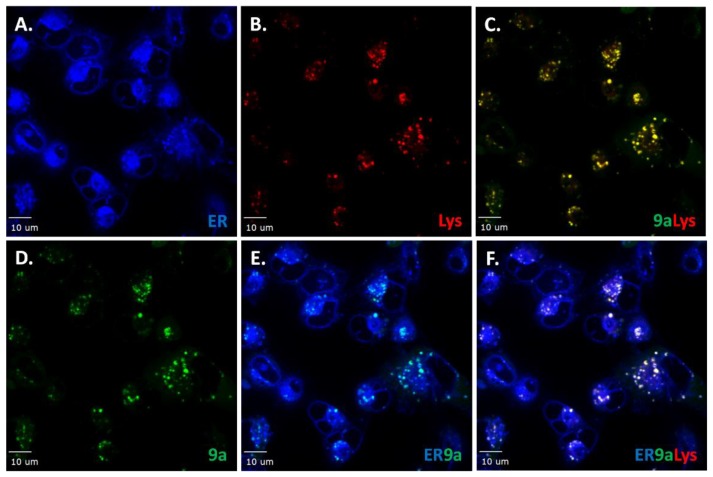
Representative images of co-localization studies of probe **9a** with organelle fluorescent stains in live cells. **A.** ER tracker Blue/White (blue). **B.** Lysosome tracker (red). **C.** Merged image of lysosomal and probe **9a** staining. **D.** Probe **9a** (2 µM, green). **E.** Merged image of ER tracker and probe **9a** staining. **F.** Merged image of ER tracker, Lysosome tracker and probe **9a** staining. Scale bar: 10 μm.

To gain further information regarding intracellular compound localization, studies were extended to include a marker for the ER (Figure 8). The staining pattern for the individual probes are shown in Figure 8A (ER), Figure 8B (Lysosome) and Figure 8D (probe **9a**). Co-localization of ER with probe **9a** (Figure 8C, yellow specks) and lysosome tracker was observed as seen in Figure 7. Co-localization of probe **9a** with ER-Tracker Blue/White (turquoise color in Figure 8E) was also observed. Moreover, when all three channels were merged, white specks were observed, indicating co-localization of probe **9a** with cellular structures that are stained by both ER and lysosomal marker dyes (Figure 8F). The data show either partial compound accumulation in the ER, while lysosomal structures are removing probe **9a** bound to the ER or active probe **9a** removal by lysosomal action before the compound can become active in the ER. These mechanisms provide a plausible explanation for the lower potency of compound **9** compared to compound **10**. Finally, in vitro ADME (absorption, distribution, metabolism, and elimination) profiles [11] were evaluated for compounds **9** and **10** to compare with the obtained cellular data (Supplementary Material, Figure S10). The aqueous solubility properties at physiological pH (7.40) of compounds **9** and **10** were good compared to controls. Simulated gastric fluid (SGF) stability was robust for compounds **9** and **10** with t_1/2_ > 33 h. Metabolic stability studies in mouse and human liver microsomes indicate that compound **9** is rapidly degraded, while compound **10** displays t1/2 of at least 1 h in both models. However, compound **9** showed remarkable stability in both mouse and human plasma stability assay (t_1/2_ > 25 h), while compound **10** showed poor stability in mouse plasma, but moderate stability in human plasma (t_1/2_ < 1 h, t_1/2_ > 12 h respectively). PAMPA assay indicated favorable permeability properties for both compounds, but caco-2 permeability assay suggests that compound **9** undergoes efflux (as the efflux ratio, B2A/A2B is closer to 2) while no efflux is suspected for compound **10** (B2A/A2B < 1) (Supplementary Material, Figure S10). The combined findings indicate that the steric-effects of the hydroxysuccinate appendage of compound **10** renders it less prone to degradation by lysosomal activity or other competing cellular removal mechanisms. 

## 3. Discussion

The combined studies indicate that natural products continue to play an important role in drug discovery. Herein, several natural products were identified to have significant cytotoxicity against aggressive ALL cellular models by affecting cell cycle and inducing cell death via caspase activation, cell cycle arrest, and apoptosis. 

Furthermore, this study highlights potential mechanistic processes responsible for the bioactive properties of cephalotaxine, compound **9**. Protein synthesis assays indicate that both compounds **9** and **10** inhibit protein synthesis, however compound **9** caused minimal cell death when compared to compound **10**. Compound **10** acts only on the initial step of protein translation and does not inhibit protein synthesis from mRNAs that have already commenced translation, unlike peptidyl transferase inhibitors such as cycloheximide (CHX), which inhibits peptide formation on mRNAs that are actively translated [34,35]. Previous medicinal chemistry campaigns [38,39,40,41,42] evaluating compound **10** had made observations that compound **9** was less potent than compound **10**. However, it was speculated that the elaborated ester side chain of compound **10** was required for interacting with the target. Co-crystallization studies of compound **10** with the large ribosomal subunit from H. marismortui, a member of the Halobacteriaceae family, have shown that protein translation is halted by preventing the initial elongation step of protein synthesis via interaction with the ribosomal A-site [42]. While both core and side chain contribute to the interactions, an important interaction of the side chain is its hydrophobic interaction with the base of U2506, which appears to lock the drug in its binding site in this model. The amine, which is protonated under physiological conditions forms a hydrogen bond with the carbonyl of C2452 [42]. This interaction should be feasible for compound **9**. However, a synthesized BODIPY-cephalotaxine probe **9a** demonstrates that while cephalotaxine (compound **9**) localized to the ER to inhibit protein synthesis, the probe **9a** also co-localized with the lysosome. Thus, this provides a potential mechanism for rapid removal of compound **9** via lysosome, which would render it less effective at inducing cell death. 

## 4. Materials and Methods 

### 4.1. Cell Culture 

All human cell lines were incubated at 37 °C in a 5% CO_2_ atmosphere and maintained under sterile conditions [43]. Cells were tested for Mycoplasma (Lonza, Alpharetta, GA, USA) using the manufacturer’s conditions prior to experiments. Cell lines were purchased from American Type Culture Collection (ATCC, Manassas, VA, USA) or Leibniz-Institute Deutsche Sammlung von Mikroorganismen und Zellkulturen GmbH (DSMZ, Braunschweig, Germany) and cultured without antibiotics unless stated. Leukemia cells were cultured in RPMI and supplemented with 10% fetal bovine serum (FBS, Hyclone, Logan, UT, USA). SUP-B15, and KOPN-8 lines were cultured in RPMI supplemented with 10% FBS (Hyclone), 1% GlutaMAX™, 1% Pen/Step, and 0.1% β-mercaptoethanol. BJ cells were cultured in EMEM media supplemented with 10% FBS (Hyclone). Breast cancer cells (MDA-MB-231 & SUM149) were cultured in DMEM and Ham’s F12 respectively, supplemented with 10% FBS, 1% GlutaMAX™, 1% Pen/Step, and 2 µM cortisol/1 µg/mL insulin for SUM149. Both cells were grown to 80% confluence densities as recommended by ATCC. PBMCs were supplemented with concanavalin-A (5 μg/mL) and IL-2 (50 U/ml). To test general cytotoxicity, the following cell numbers were used for 384 well plates: KOPN-8 (ACC 552, infant human B cell precursor acute lymphoblastic leukemia with MLL-MLLt1/ENL fusion, 1000 cells/well), SUP-B15 (ACC389, human B cell precursor acute lymphoblastic leukemia of pediatric second relapse carrying the ALL-variant (m-bcr) of BCR-ABL1 fusion gene (e1-a2), 1600 cells/well), NALM-06 (DSMZ ACC128, non-T/non-B ALL at relapse with P15INK4B and P16INK4A deletions, 1200 cells/well), UoC-B1 (pediatric BCP-ALL at second relapse with TCF3/E2A-HFL fusion, 1000 cells/well), BJ (CRL-2522, normal human foreskin fibroblast cells, 400 cells/well) and PBMCs (periphery blood monocyte cells from healthy donors, IBC#BDC046, 10,000 cells/well). 

### 4.2. Natural Product Compound Library

A 5000-fraction library was assembled from 48,000 natural product fractions and 2000 pure natural products in the collection of St. Jude Children’s Research Hospital which includes bioactive natural products purchased from Sigma-Aldrich (St. Louis, MO, USA), ChromaDex (Longmont, CO, USA), Med Chem Express (Monmouth Junction, NJ, USA), and ChemBridge (San Diego, CA, USA). All fractions were dissolved in DMSO at 2 mM based on mass spectrometry, arrayed in 384 polypropylene plates and stored at −20 °C. 

### 4.3. CellTiter-Glo Viability Assay (CTG)

Cytotoxicity evaluation was performed using the CellTiter-Glo Luminescent Cell Viability Assay kit (G7570, Promega, Madison, WI, USA), according to the manufacturer’s instructions. Briefly, the cell concentrations used were experimentally determined to ensure logarithmic growth during the 72-h duration of the experiment, and avoid adverse effects on cell growth by DMSO exposure. 1 × 10^3^–4.8 × 10^3^ or 4 × 10^2^–1.2 × 10^3^ cells/well were seeded in 96 or 384-well white flat-bottomed plates (3610 or 8804BC, Corning, Corning, NY, USA) in 100 µL or 30 µL/well, respectively. The plates were incubated at 37 °C in 5% CO_2_ for 24 h before drugging. Test compounds (10 mM in DMSO) in nine 3-fold serial dilutions were dispensed via pintool (Biomek liquid handler, Beckman, Indianapolis, USA) to assay plates. The final concentration of DMSO was 0.3% (*v*/*v*) in each well. The positive controls included staurosporine (10 μM), and gambogic acid (10 μM). The plates were incubated for 72 h at 37 °C in 5% CO_2_, then quenched with CellTiter-Glo^®^ (Promega, Madison, WI, USA, 50 µL/96 or 30 µL/384), centrifuged at 1000 rpm for 1 min and incubated at RT for 20 min. Luminescence was recorded with a plate reader (Envision, Perkin Elmer, Waltham, MA, USA). The mean luminescence of each experimental treatment group was normalized as a percentage of the mean intensity of untreated controls. EC_50_ values were calculated by Pipeline Pilot software (Accelrys, Enterprise Platform, San Diego, CA, USA).

### 4.4. Annexin V-FITC Apoptosis and Cell Cycle

The samples were probed with AnnexinV-FITC (Roche/Boehringer Mannheim, Indianapolis, IN, USA) according to the manufacturer’s instructions. KOPN-8 or SUB-P15 cells were plated (1.00 × 10^6^ cells/plate) and incubated for 12 h and incubated at 37 °C. Then, cells were treated with compounds or controls for 24 h. Cells were stained with AnnexinV-FITC, PI and the staining profiles were determined with FACScan and Cell-Quest software. For cellular DNA content, the same cell treatment as above was performed. Then, cells were fixed in cold 75% ethanol, treated with RNase and then stained with PI solution (50 µg/mL). Cell cycle distribution was analyzed with the FACSCalibur analyzer (BD Biosciences, Franklin Lakes, NJ, USA) and Cell-Quest software. The percentage of DNA content at different phases of the cell cycle was analyzed with ModFit-software (version 5.0, Verity Software House, Topsham, ME, USA).

### 4.5. ApoTox-Glo^TM^ Triplex Assay

Cells (9.5 × 10^4^ cells/well in 75 µL) were dispensed in 96-well black flat bottom (8807BC, Corning) plates. The cells were incubated for 12 h at 37 °C and treated with compounds (25 µL) for 36 h. DMSO was used as a negative control and camptothecan was used as the positive control. Then, the experiment was stopped by adding the Viability/Cytotoxicity Reagent, and briefly mixed by orbital shaking (300–500 rpm for ~30 s). Plates were incubated for 30 min at 37 °C and fluorescence was measured at the wavelength 400Ex/505Em for viability and 485Ex/520Em for cytotoxicity in an Envision plate reader (Perkin Elmer). Finally, the Caspase-Glo® 3/7 Reagent was added, and the plates were mixed by orbital shaking (300–500 rpm for ~30 s), followed by an additional 30 min incubation at RT. Luminescence was recorded with a plate reader (Envision, Perkin Elmer) to capture caspase 3/7 activation and determine apoptosis induction.

### 4.6. Protein Synthesis in Cell-Click Assay

Biovision’s EZClick™ protein synthesis monitoring assay kit (EZClick™ Global Protein Synthesis Assay Kit, Catalog # K715-100, Milpitas, CA, USA) was used according to manufacturer’s protocol. The assay was conducted in 8-well chambered coverslips (ibidi GmbH µ-slide # 80826, Martinsried, Germany). Cells were plated at 2 × 10^4^ per well and incubated at 37 °C for 12 h. Then, the cells were treated with compounds for 1.5 h and processed and stained according to protocol (representative images shown in Figure 4). Protein synthesis activity is shown in red, DNA staining is shown in green. Cycloheximide (CHX) was used as a positive control and DMSO as the negative control. Images shown were taken with a Marianas CSU-X spinning disk confocal imaging system (3i, Denver, CO, USA) configured with a Zeiss Axio Observer microscope (Carl Zeiss Inc., Thornwood, NY, USA) with diode lasers, at 63× magnification and resolution of 512 × 512 pixels (3i). For each field of view, an image stack consisting of 20 optical sections were taken at 63× magnification. The total intensity of red and green staining was quantified for the entire image stack. Six fields of view were analyzed per condition. Images shown represent a single optical section for each field of view. For each stack, the red intensity (protein synthesis) was adjusted to the green intensity (DNA content) in each image. The numbers from six images were used to calculate the average and standard deviation for each condition. Numbers were normalized to the negative control value arbitrarily set to one. The Slide viewer 6 software package was used for rendering and analysis of the images. 

### 4.7. Probe ***9a*** Evaluation

Co-localization studies were conducted in 8-well chambered coverslips (ibidi GmbH µ-slide # 80826) for MDA-MB-231. Coverslips were first coated with 0.1% Gelatin for 30 min for better cell adherence. Cells were then plated in phenol red free medium at a density of 4 × 10^4^ cells per well and incubated at 37 °C overnight. Then the cells were treated with 1–10 µM of **9a** probe and/or organelle tracker for 1 h at the following final concentrations: 1 µM ER-Tracker™ Blue-White DPX (E12353, Invitrogen); 100 nM LysoTracker™ Red (L7528, Invitrogen, Carlsbad, CA, USA); 250 nM MitoTracker™ Deep Red FM (M22426, Invitrogen), Invitrogen). Thirty minutes after addition of the probe and tracking dyes, nuclear stain Hoechst33342 (H3570, Invitrogen) was added to samples not stained with ER-Tracker Blue/White at a final concentration of 500 nM. After incubation for a total of 1 h, cells were washed twice with fresh medium, followed by live imaging on a Marianas CSU-X spinning disk confocal imaging system configured with a Zeiss Axio Observer microscope with diode lasers, at 63× magnification and resolution of 512 × 512 pixels (3i).

### 4.8. Statistical Analysis

Statistical analysis of data was performed using GraphPad Prism (Version 7.0 San Diego, CA, USA) and Microsoft Excel software (Office 2010, Microsoft Corp., Redmond, WA, USA). The statistical methods used were repeated-measures analysis of variance and Tukey’s test for paired data when appropriate; a *p* value less than 0.025 was considered statistically significant and no statistical significant was depicted by ns. Standard error bars represented the standard deviation of the mean (± SD).

## 5. Conclusions

In summary our study demonstrates that natural products continue to provide potential hits against aggressive high risk ALL. A screen of a focused natural product fractions library identified several active alkaloids and steroidal compounds against high-risk ALL cellular models. The alkaloids **8**, **9**, and **10** (chelerythrine, cephalotaxine, homoharringtonine) and steroidal compounds **12** and **13** (ecdysone, and muristerone A) were validated by viability and apoptotic assays as cytotoxic and cytostatic agents, respectively. Our studies indicate these natural products (compound **8**–**13**) have promising therapeutic index in normal cells (BJ and PBMCs), therefore further development is warranted to improve their efficacy against these cancer subtype cell lines. Solid tumor cell models (breast cancer) were utilized for microscopy studies (not feasible in suspension cell lines) to better understand the properties of compounds **9** and **10**. Our study shows that both compounds **9** and **10** inhibit protein synthesis, but compound **10** is more efficacious at inducing cell death. By synthesizing a tool compound, probe **9a**, we demonstrate for the first time that compound **9** is rapidly removed via lysosome activity, limiting its cytotoxic effects in the cell. Thus, providing insight into the mechanism of these alkaloid compounds, and highlighting potential sites for future derivatization to improve the activity/subcellular stability of this family of natural products against high risk ALL models. 

## Figures and Tables

**Table 1:** CTG viability assay (CellTiter-Glo, 72 h) ^1^, which quantitates the amount of ATP present to determine cell viability. Hit compounds (**1**–**13**) were evaluated against several ALL cell lines and non-cancerous cell lines (BJ/PBMC cells) to determine therapeutic index.

	SUP-B15 (EC_50_ µM)	KOPN-8 (EC_50_ µM)	NALM-06 (EC_50_ µM)	UoC-B1 (EC_50_ µM)	BJ (EC_50_ µM)	PBMC (EC_50_ µM)	TI (PBMC/SUP-B15)
**1**	0.1201	0.41 ± 0.05	0.41 ± 0.15	0.0401 ± 0.01	>38.8788	>38.8788	>316
**4**	38 ± 6.1	32.1 ± 3.5	18.3 ± 2.5	>23.3766	>46.7532	>86.7532	>2.25
**5**	29 ± 3.7	32 ± 7.6	8.3832 ± 1.8	17.28 ± 3.8	>43.2900	>43.2900	>1.48
**6**	9.83 ± 0.5	0.1602 ± 0.05	18.45 ± 3.1	>21.6450	>43.2900	>43.2900	>4.3
**7**	6.05 ± 0.8	4.0732 ± 0.45	3.7229 ± 0.5	2.88 ± 1.1	>51.9481	28.0668	4.6
**8**	2.77 ± 0.36	0.8208 ± 0.22	1.9 ± 0.2	1.96 ± 0.28	29.8382	7.8914	2.8
**9**	10.43 ± 0.75	23.12 ± 5.2	9.5 ± 2.7	17.6 ± 5.8	>43.2900	>83	>7.7
**10**	0.045 ± 0.005	0.0799 ± 0.01	0.0322 ± 0.01	0.0129 ± 0.002	>43.2900	6.3499	140
**11**	29 ± 6	>43.2900	>43.2900	>21.6450	>43.2900	>43.2900	>1.48
**12**	20.1 ± 3.2	>43.2900	>43.2900	>21.6450	>43.2900	>43.2900	>2.15
**13**	9.5 ± 2.1	>43.2900	0.518 ± 0.15	>21.6450	>43.2900	>43.2900	>4.5
**14**	17.2 ± 4.5	>43.2900	>43.2900	>21.6450	>43.2900	>43.2900	>2.5

^1^ Table represents mean ± SEM of triplicates. Analysis via Pipeline pilot or GraphPad Prism program.

## Supplementary Materials

The supplementary materials are available online.
