# Supplementary material for: Cytostatic and Cytotoxic Natural Products against Cancer Cell Models
Source: Molecules. 2019 May 26;24(10):2012. doi: 10.3390/molecules24102012 (PMC6571673; doi:10.3390/molecules24102012)

Supporting Information

Cytostatic and Cytotoxic Natural Products against Cancer Cell Models

Taotao Ling[1], Walter H. Lang[1], Julie Maier[1], Marizza Quintana Centurion[2] and Fatima Rivas[1,*]

Department of Chemical Biology and Therapeutics, St. Jude Children's Research Hospital. 262 Danny Thomas Place. Memphis, TN 38105-3678, Unites States.

Dirección de Investigación Biológica/Museo Nacional de Historia Natural del Paraguay, Casilla de Correo 19.004. Sucursal 1, Campus UNA. 169 CDP San Lorenzo, Central XI, Paraguay

RECEIVED DATE (automatically inserted by publisher); Fatima.rivas@stjude.org

Contents of Supporting Information

Page

I. **General experimental information** ……………………………………………………3
Figure S1. Representative images of Z-prime for CTG assay ……………………………………5
Figure S2. Representative scatterplot of activity values from screen…………………………………5
Figure S3. Representative graphs of CTG viability assay in 384 well plates ……………………………6
Figure S4. Representative graphs of CTG viability assay in 96 well plates ……………………………7
Figure S5. Representative images of Annexin V-FITC plots for KOPN-8 cells ………………………8
Figure S6. Representative images of Annexin V-FITC plots for SUP-B15 cells ………………………8
Figure S7. Representative Modfit graphs of KOPN-8 cells ……………………………………………9
Figure S8. Representative Modfit graphs of SUP-B15 cells ……………………………………………10
Figure S9. Representative images of protein synthesis assay with DAPI stain ………………………11
Figure S10. In vitro ADME of compounds **9** and **10** ……………………………………………………12
Figure S11. ^{1}H NMR Spectra of compound **9** ……………………………………………………13
Figure S12. ^{13}C NMR Spectra of compound **9** ……………………………………………………13
Figure S13. ^{1}H NMR Spectra of compound **9a**……………………………………………………14
Figure S14. ^{13}C NMR Spectra of compound **9a** ……………………………………………………14

I) General Chemistry Information

All manipulations were carried out under inert gas atmosphere unless otherwise noted. Anhydrous tetrahydrofuran (THF), diethyl ether (Et_2O), dichloromethane (CH_2Cl_2), toluene ($PhCH_3$), acetonitrile (CH_3CN), methanol (MEOH), and dimethylformamide (DMF) were obtained from solvent drying system. Reagents of the highest available quality were purchased commercially and used without further purification unless otherwise stated. Title compounds were purified by flash column chromatography using E. Merck silica gel (60, particle size 0.040–0.063 mmol) or Biotage Isolera Four with normal-phase silica gel. Reactions were monitored by thin-layer chromatography (TLC) on 0.25 mmol E. Merck silica gel plates (60F-254), using UV light for visualization and an ethanolic solution of anisaldehyde, or PMA, CAM solutions and heat as developing agent. Reactions were also monitored by using Agilent 1100 series LCMS and low-resonance electrospray ionization (ESI) model with UV detection at 250 nm. The structures of the synthesized compounds were confirmed by ^{1}H and ^{13}C-NMR that were recorded on 400/or 500 MHz Bruker AVANCE III HD NMR. Chemical shifts were reported as ppm relative to the solvent residual peak ($CHCl_3$: 7.26 ppm for ^{1}H, 77.2 ppm for ^{13}C; acetone-d_6: 2.05 ppm for ^{1}H, 29.9 ppm for ^{13}C; D_2O: 4.80 ppm for ^{1}H; DMSO d_6: 39.5 ppm for ^{13}C). Data are reported as follows: chemical shifts, multiplicity (s = singlet, d = doublet, t = triplet, q = quartet, quint = quintet, m = multiplet, br = broad), coupling constant (Hz), and integration. High resolution mass spectra (HRMS) were recorded on an Agilent ESI-TOF (time of flight) mass spectrometer using MALDI (matrix-assisted laser desorption ionization) or ESI (electrospray ionization) or on a Waters Xevo G2 Q-ToF mass spectrometer. Compounds were analyzed by using electrospray ionization in positive-ion mode. The purity of the synthesized compounds was determined on a Waters ACQUITY UPLC-PDA-ELSD-MS system using a C_{18} reverse phase column and 0.1% formic acid/water - 0.1% formic acid/acetonitrile as the solvents. All synthesized compounds were at least 95% pure based on analytical HPLC and NMR. Chemical yields refer to purified compounds (^{1}H-NMR).

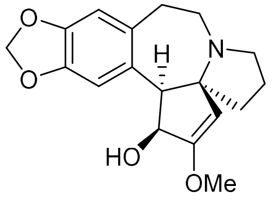

(-)-Cephalotaxine, compound **9.** Purchased from MCE, cat# HY-N0838; ^{1}H NMR (500 MHz, Chloroform-*d*) δ 6.66 (d, *J* = 15.6 Hz, 2H), 5.90 (q, *J* = 1.6 Hz, 1H), 4.93 (s, 1H), 4.77 (dd, *J* = 9.4, 3.5 Hz, 1H), 3.73 (s, 1H), 3.68 (d, *J* = 9.4 Hz, 1H), 3.39 – 3.26 (m, 1H), 3.07 (dd, *J* = 8.6, 4.9 Hz, 1H), 2.95 – 2.86 (m, 1H), 2.62 – 2.52 (m, 1H), 2.35 (dd, *J* = 14.5, 7.0 Hz, 1H), 2.00 (s, 1H), 1.87 (td, *J* = 8.3, 4.0 Hz, 1H), 1.74 (d, *J* = 4.9 Hz, 2H), 1.55 (s, 6H). ^{13}C NMR (126 MHz, $CDCl_3$) δ 160.82, 147.30, 146.49, 134.67, 128.36, 113.05, 110.76, 101.33, 98.03, 77.67, 77.62, 77.41, 77.16, 73.68, 70.96, 58.41, 57.63, 54.33, 49.03, 44.04, 32.08, 20.70, 0.40.

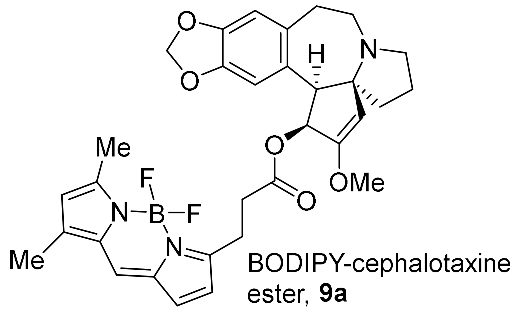

BODIPY-cephalotaxine ester, **9a**

Cephalotaxine Probe, 9a. ^{1}H NMR (500 MHz, Chloroform-d) δ 7.04 (s, 1H), 6.82 (d, J = 4.1 Hz, 1H), 6.63 (s, 1H), 6.59 (s, 1H), 6.09 (s, 1H), 6.01 (d, J = 4.1 Hz, 1H), 5.89 – 5.84 (m, 2H), 5.04 (s, 1H), 3.78 (d, J = 9.4 Hz, 1H), 3.71 (s, 3H), 3.24 (ddd, J = 14.9, 12.6, 7.7 Hz, 1H), 3.08 (s, 1H), 2.89 (t, J = 8.3 Hz, 3H), 2.63 – 2.52 (m, 3H), 2.45 – 2.36 (m, 1H), 2.23 (s, 3H), 2.17 (dt, J = 15.8, 7.8 Hz, 1H), 1.93 – 1.85 (m, 1H), 1.82 – 0.75 (m, 7H); 13C NMR (126 MHz, CDCl3) δ 171.33, 171.16, 159.99, 157.46, 146.63, 145.56, 143.40, 135.01, 133.71, 133.23, 128.16, 128.02, 123.64, 120.21, 116.47, 113.28, 109.90, 100.72, 100.18, 74.70, 70.59, 60.39, 57.24, 56.44, 53.88, 53.41, 48.59, 43.32, 32.79, 31.33, 29.69, 23.31, 21.05, 20.20, 14.90, 14.19, 11.28. HRMS (ESI) calcd. for C32H35BF2N3O5 [M+H]+ 590.2638, found 590.2641.

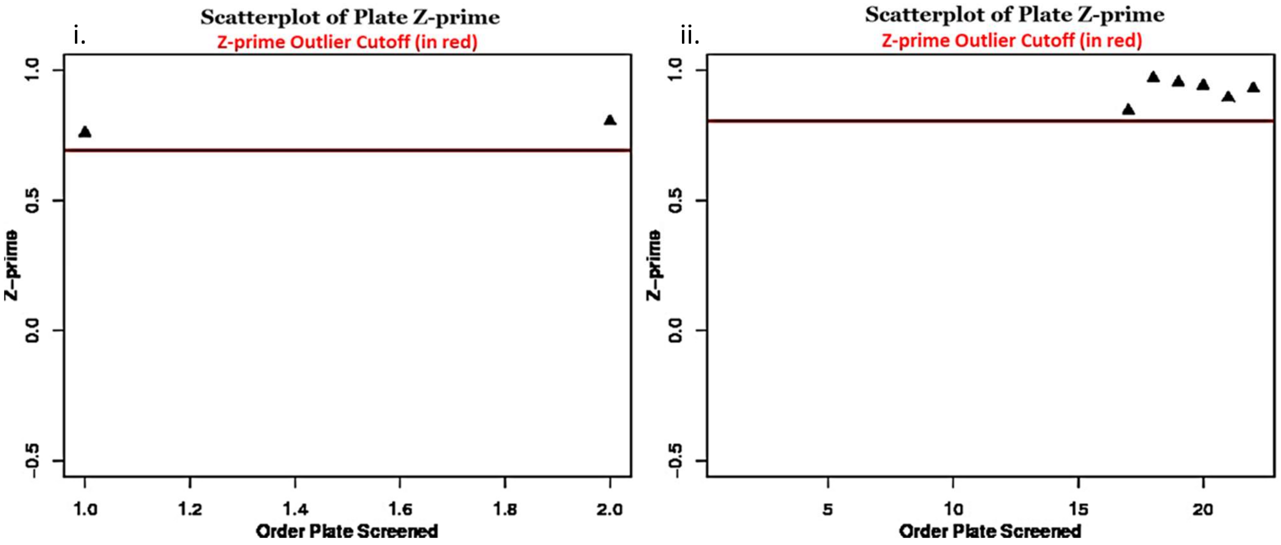

Figure S1. Representative images of z-prime for CTG assay via Pipeline Pilot Software (Accelrys, Enterprise Platform, CA, USA). i. PBMCs assay plates with Donor 1 and Donor 2 cells respectively. ii. SUP-B15 cell plates (2 sets of triplicates) for 2 drug plates.

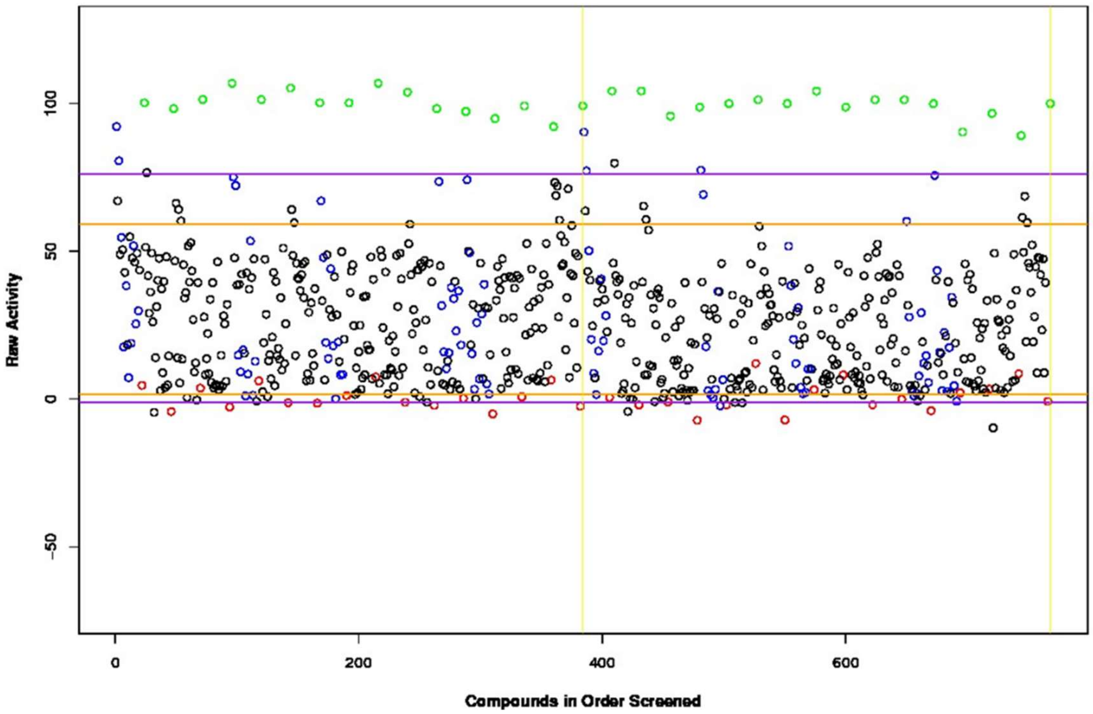

Figure S2. Representative scatterplot of activity values from screen. Positive control (in green, staurosporine, gambogic acid, and a cytotoxic quinoline generated in house), negative control (in red), inactive compounds (in black), active compounds (in blue) 95th activity quantile (in orange), 99th activity quantile (in purple).

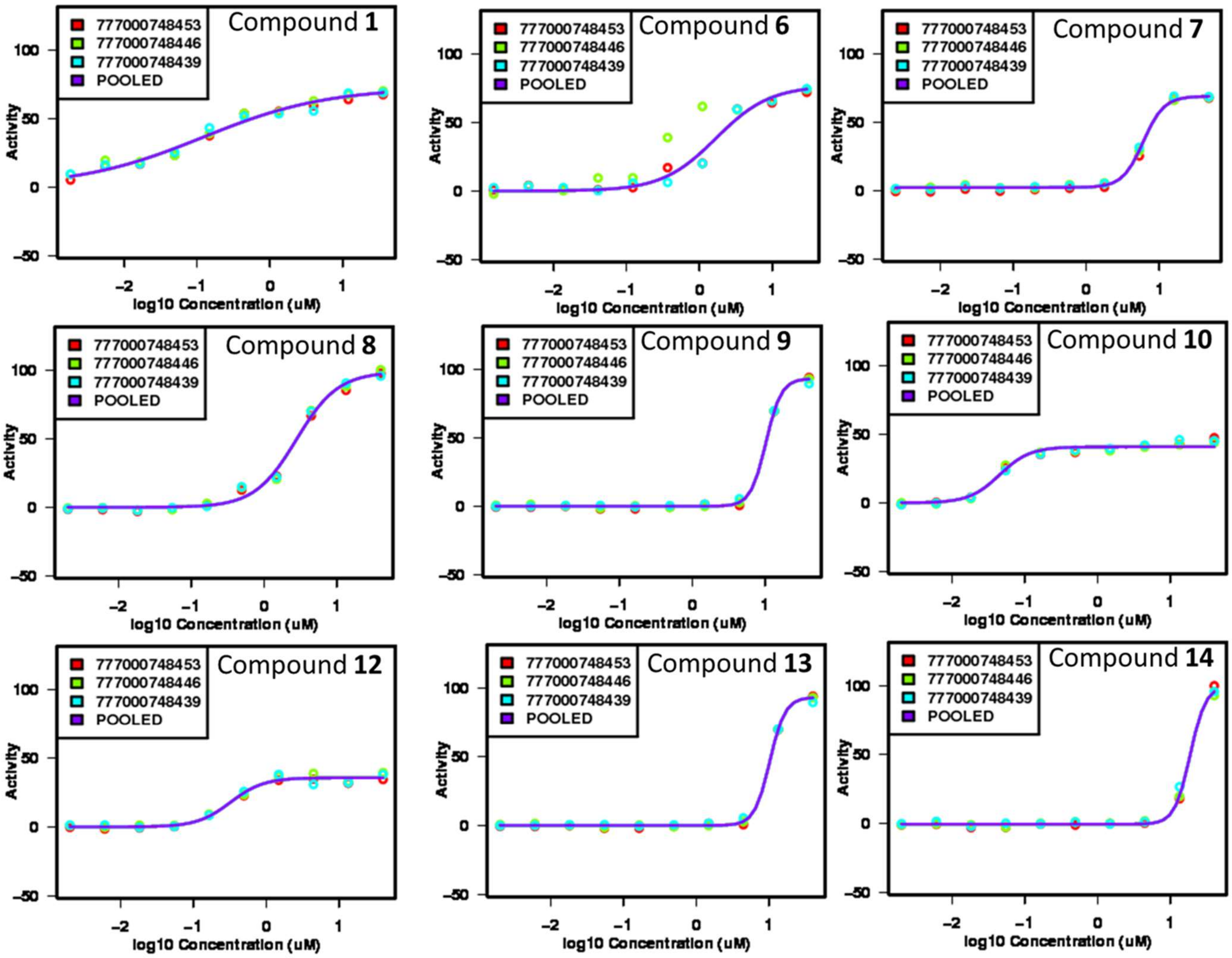

Figure S3. Representative graphs of CTG viability assay of SUP-B15 data (384 well plates) analysis via by Pipeline Pilot software (Accelrys, Enterprise Platform, CA, USA).

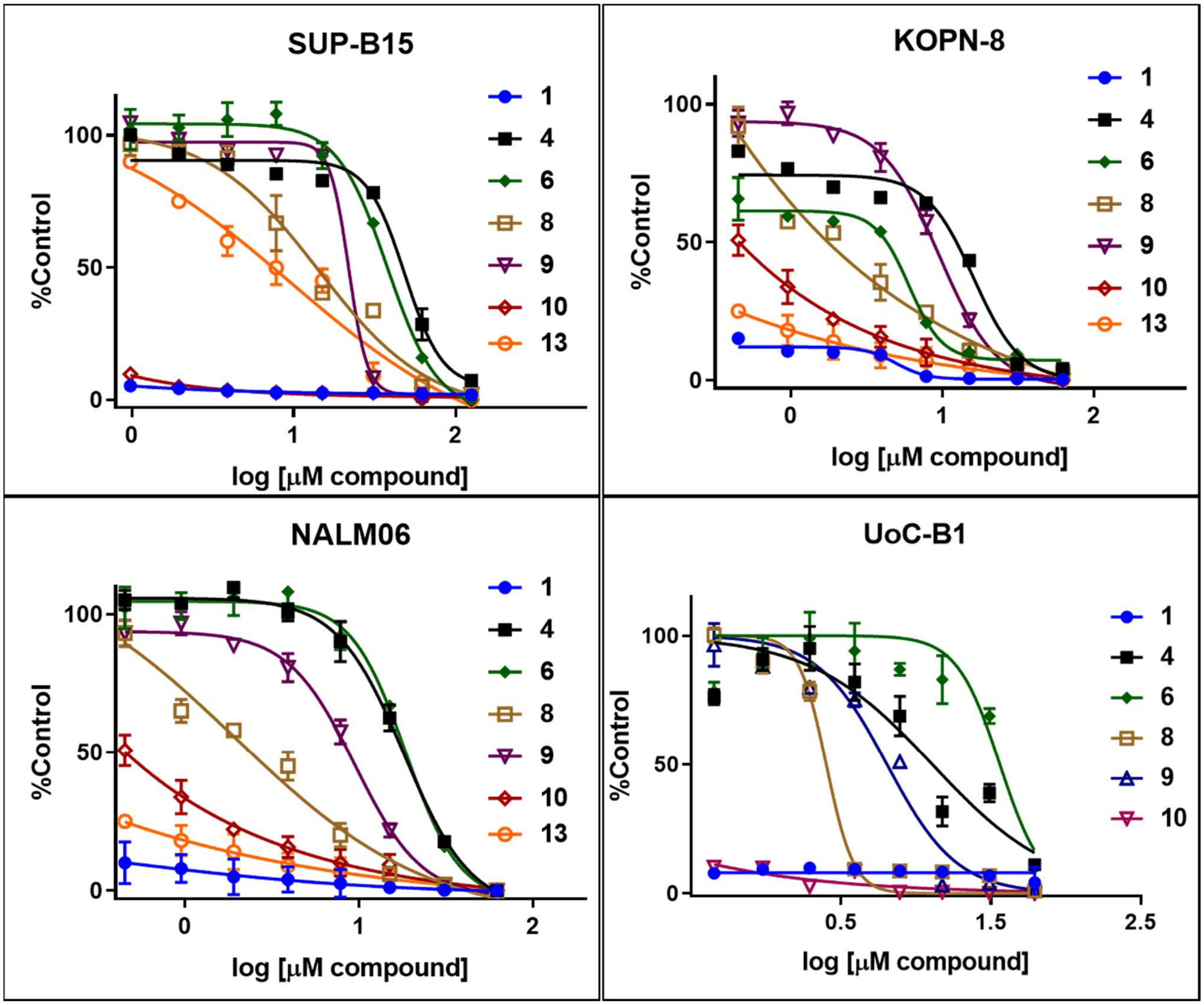

Figure S4. Representative graphs of CTG viability assay for 96 well plates via GraphPad Prism (Version 7.0 San Diego, CA).

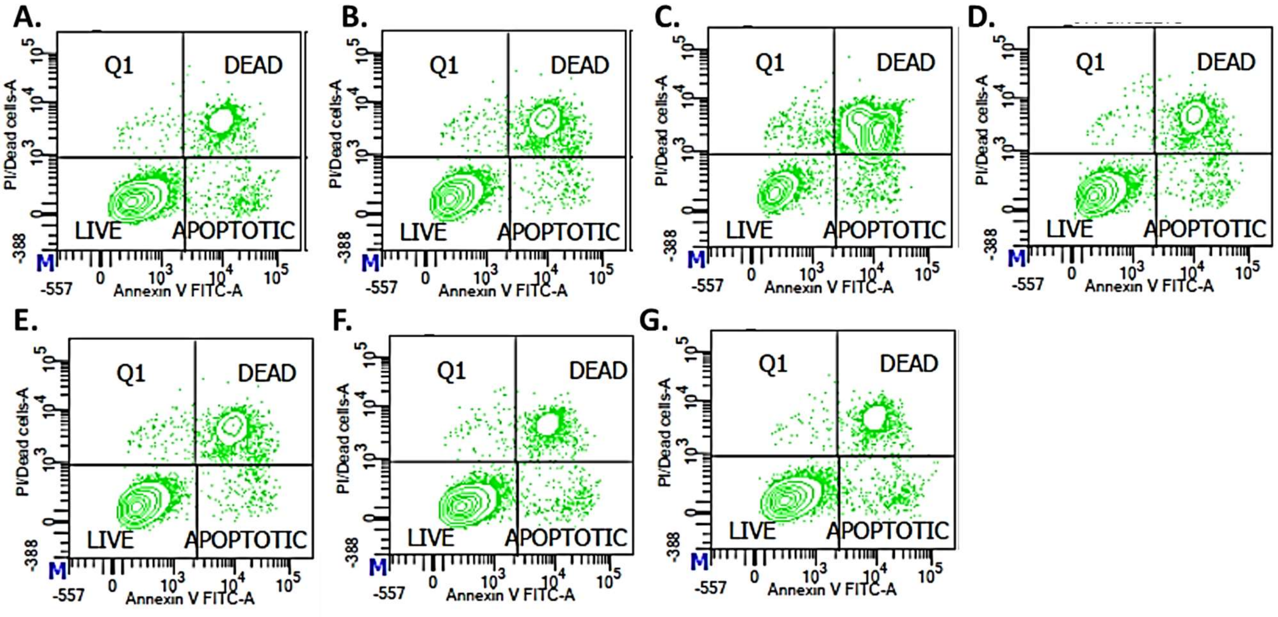

Figure S5. Representative images of Annexin V-FITC plots for KOPN-8 cells. **A.** DMSO. **B.** Silvestrol (5 μM). **C.** Compound **8** (5 μM). **D.** Compound **9** (10 μM). **E.** Compound **10** (5 μM). **F.** Compound **12** (10 μM). **G.** Compound **13** (10 μM). Three cell populations were measured: viable cells (negative for Annexin V-FITC and PI staining), early apoptotic cells (annexin V-FITC–positive, PI-negative), and late apoptotic or dead cells (positive for annexin V-FITC and PI).

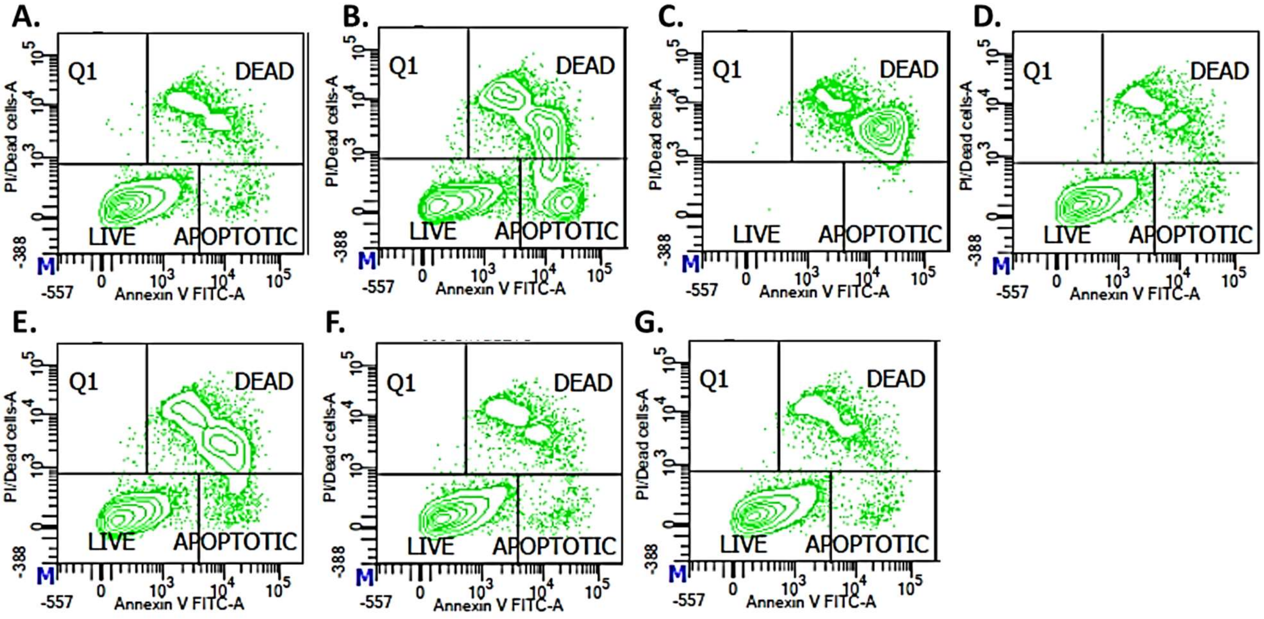

Figure S6. Representative images of Annexin V-FITC plots for SUP-B15 cells. **A.** DMSO. **B.** Silvestrol (5 μM). **C.** Compound **8** (5 μM). **D.** Compound **9** (10 μM). **E.** Compound **10** (5 μM). **F.** Compound **12** (10 μM). **G.** Compound **13** (10 μM).

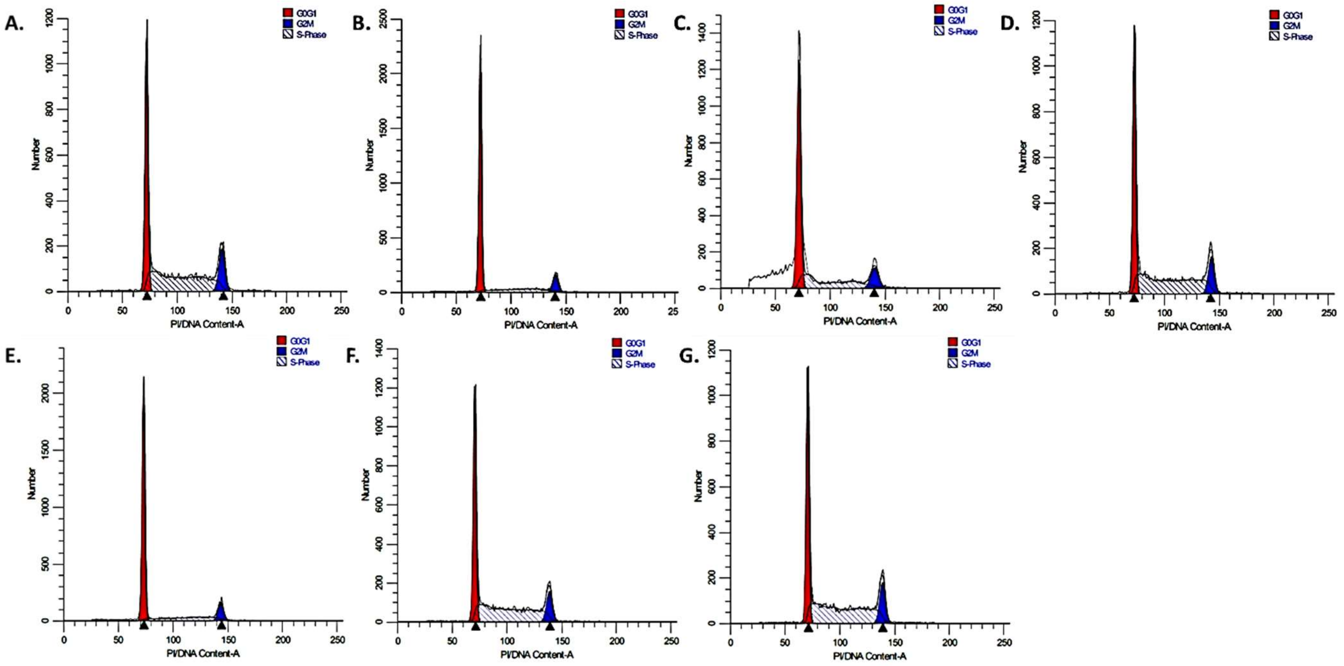

Figure S7. Representative Modfit graphs of KOPN-8 cells, which were incubated with compounds for 24 h. Cellular DNA content was measured by flow cytometry (FACSCalibur, Becton–Dickinson). **A.** DMSO, negative control, **B.** Silvestrol (5 μM). **C.** Compound **8** (5 μM). **D.** Compound **9** (10 μM); **E.** Compound **10** (5 μM). **F.** Compound **12** (10 μM). **G.** Compound **13** (10 μM).

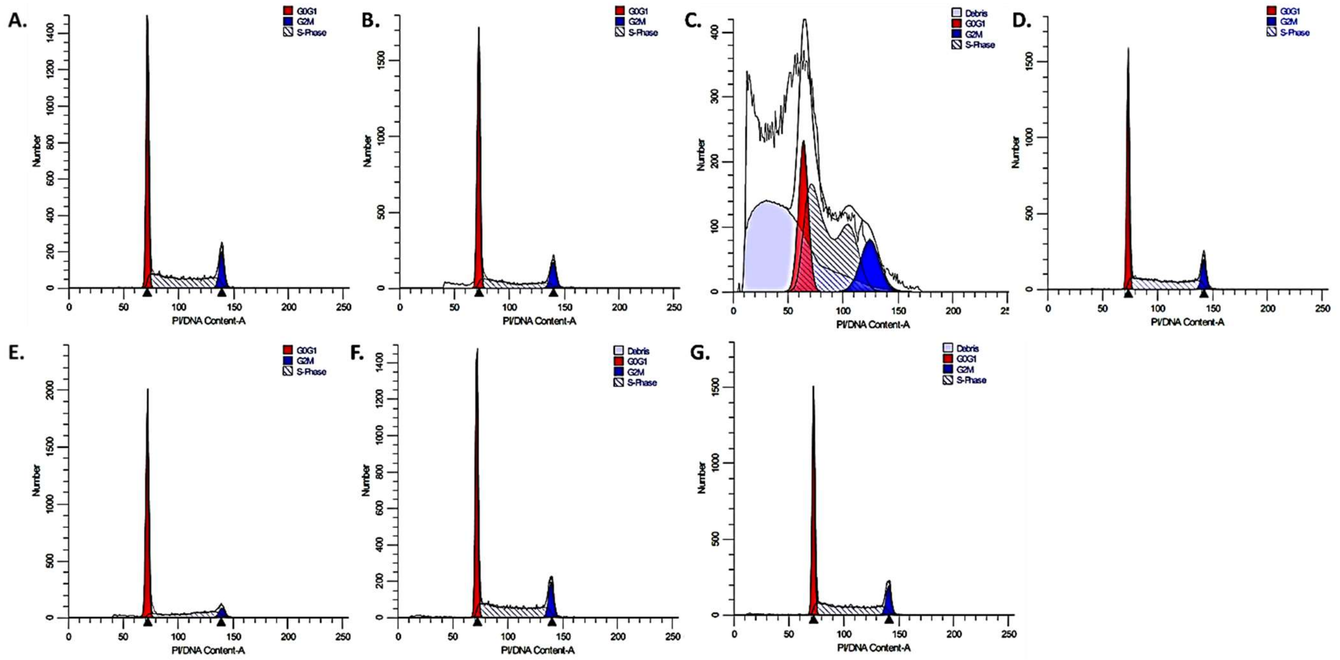

Figure S8. Representative Modfit graphs of SUP-B15 cells, which were incubated with compounds for 24 h. Cellular DNA content was measured by flow cytometry (FACSCalibur, Becton–Dickinson). **A.** DMSO, negative control, **B.** Silvestrol (5 μM). **C.** Compound **8** (5 μM). **D.** Compound **9** (10 μM); **E.** Compound **10** (5 μM). **F.** Compound **12** (10 μM). **G.** Compound **13** (10 μM).

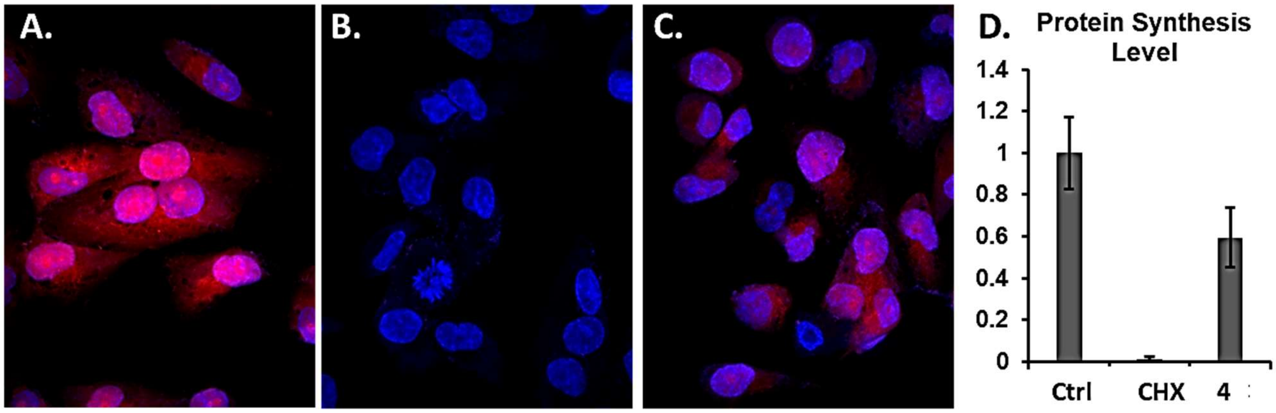

Figure S9. Representative images of 2 h incubation with compounds of interest using MDA-MB-231 cellular model. **A.** Vehicle (DMSO). **B.** Positive control CHX (1 μM). **C.** Compound **4** (5 μM). **D.** Relative quantification.

	Metabolic Stability (Mouse)			Metabolic Stability (Human)		
Compound ID	t1/2 (hr)	STD	Clint (mL/Min/Kg)	t1/2 (hr)	STD	Clint (mL/Min/Kg)
Verapamil	0.47	0.02	120.72	0.82	0.04	25.51
Compound **9**	<0.08	---	---	<0.08	---	---
compound **10**	1.16	0.05	49.28	1.68	0.24	12.37

	Plasma Stability (Mouse)		Plasma Stability (Human)	
Compound ID	t1/2 (hr)	STD	t1/2 (hr)	STD
Eucatropine	0.61	0.01	0.29	0.03
Compound **9**	25.81	3.66	37.47	5.59
compound **10**	0.36	0.01	12.53	0.69

	PAMPA				
Compound ID	pH	Avg Pe (10-6cm/s)	SD Pe	%R	SD R
Carbamazepine	7.40	139.40	21.52	36.69	3.61
Ranitidine	7.40	2.58	2.41	10.61	0.12
Compound **9**	7.40	22.09	2.98	15.87	4.57
compound **10**	7.40	28.20	4.08	18.67	1.17

Caco 2 Permeability					
Compound ID	AVG Papp A/B (nm/s)	SD Papp A/B	AVG Papp B/A (nm/s)	SD Papp B/A	Efflux Ratio (B2A/A2B)
Carbamazepine_1	154.68	2.41	143.30	27.70	0.93
Vinblastine_1	5.79	4.60	32.14	15.92	5.55
Compound **9**	29.95	10.67	58.99	29.79	1.97
compound **10**	47.41	36.75	28.48	14.14	0.60

Solubility (LC/MS)		
Compound ID	Avg. Sol (µM)	SD Sol (µM)
Carbamazepine	64.41	4.46
Ranitidine	104.53	56.78
Verapamil	57.74	0.92
Compound **9**	91.22	15.36
compound **10**	94.00	2.38

SGF Stability		
Compound ID	t1/2 (hr)	STD
Chlorambucil	10.53	0.25
Compound **9**	41.41	7.00
compound **10**	33.25	5.08

Figure S10. Representative in vitro ADME analysis of compound **9** and **10** along with control compounds.

Figure S11. ^{1}H NMR Spectra of compound **9**.

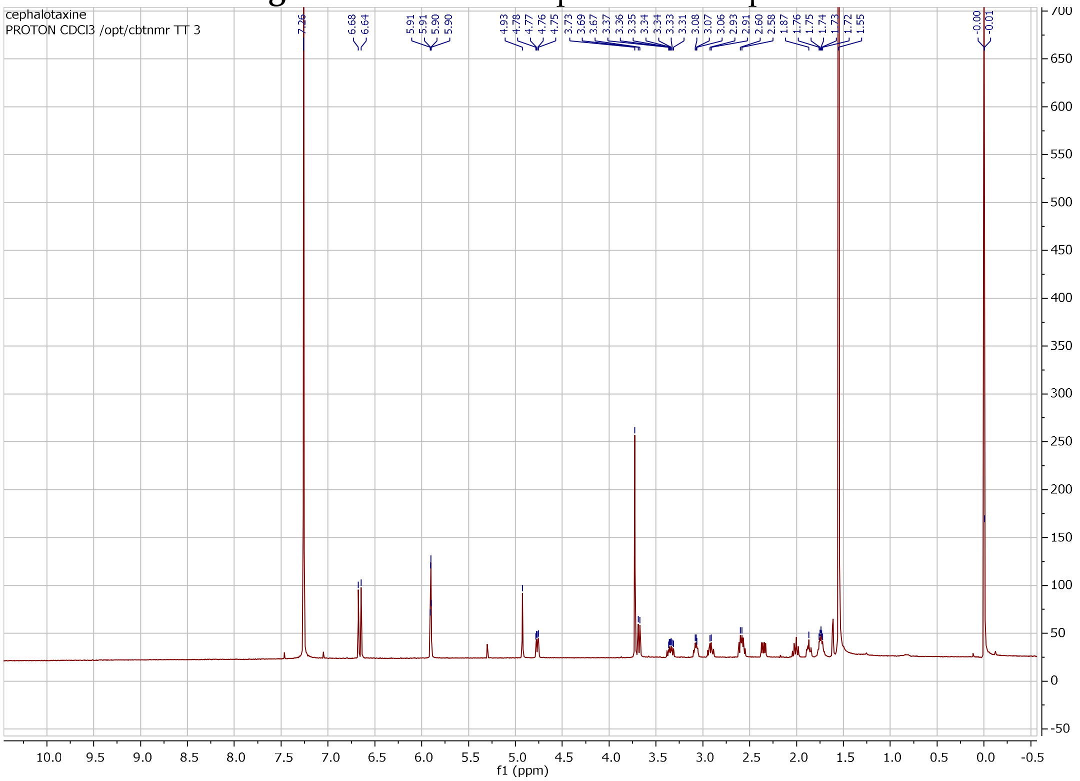

Figure S12. ^{13}C NMR Spectra of compound **9**.

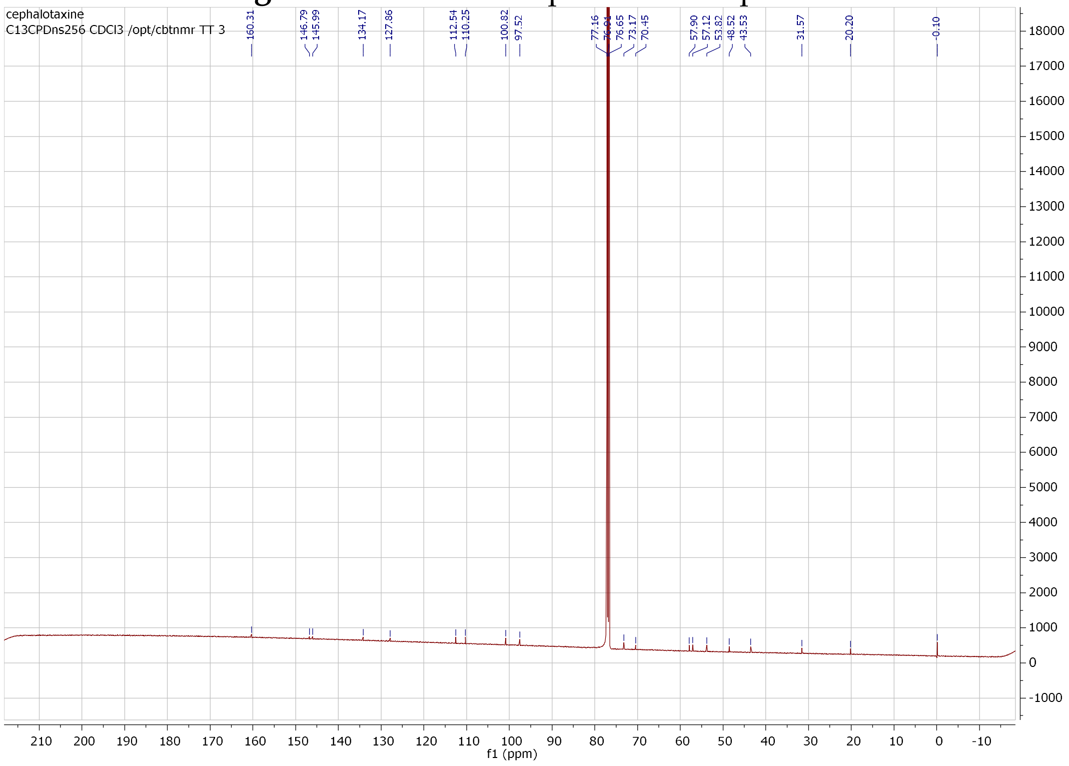

Figure S13. ^{1}H NMR Spectra of compound **9a**.

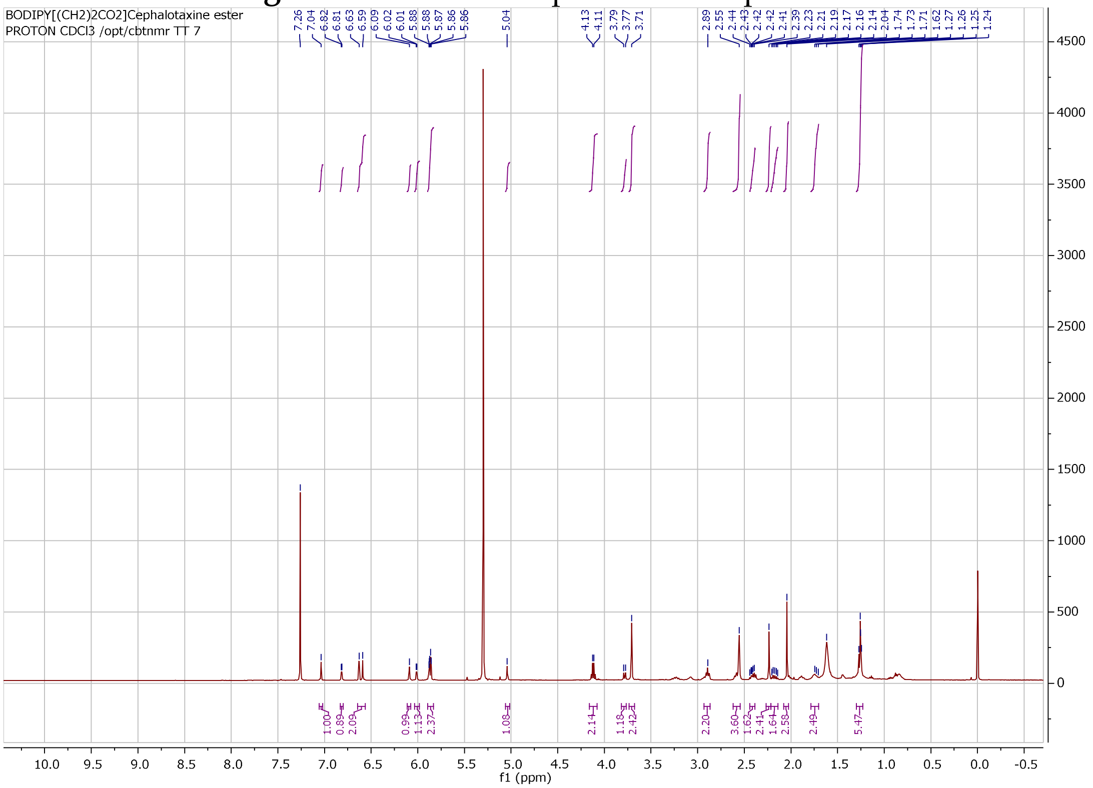

Figure S14. ^{13}C NMR Spectra of compound **9a**.

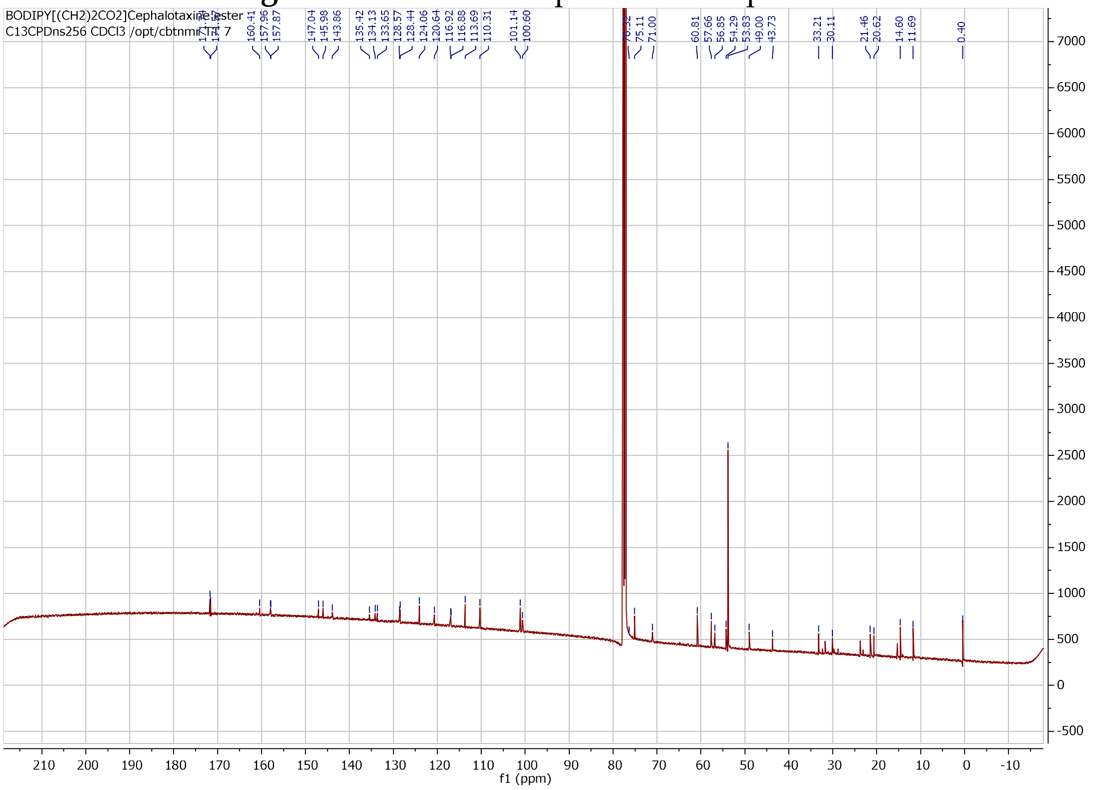

Supplement: Supplementary file 1 [file molecules-24-02012-s001.pdf]
